# Exploration of genetic diversity of *Plasmodium vivax* circumsporozoite protein (*Pvcsp*) and *Plasmodium vivax* sexual stage antigen (*Pvs25*) among North Indian isolates

**DOI:** 10.1186/s12936-019-2939-z

**Published:** 2019-09-06

**Authors:** Hargobinder Kaur, Rakesh Sehgal, Archit Kumar, Alka Sehgal, Praveen K. Bharti, Devendra Bansal, Pradyumna K. Mohapatra, Jagadish Mahanta, Ali A. Sultan

**Affiliations:** 1 Department of Medical Parasitology, Postgraduate Institute of Medical Education and Research, Chandigarh, 160012 India; 2 Department of Virology, Postgraduate Institute of Medical Education and Research, Chandigarh, India; 3 Department of Obstt. & Gynae, Government Medical College and Hospital, Chandigarh, India; 4 National Institute for Research in Tribal Health, Indian Council of Medical Research, Nagpur Road, Garha, Jabalpur, Madhya Pradesh India; 5 Department of Microbiology and Immunology, Weill Cornell Medicine-Qatar, Cornell University, Qatar Foundation-Education City, Doha, Qatar; 6 Regional Medical Research Centre, NE, Indian Council of Medical Research, Post Box no.105, Dibrugarh, Assam India; 7 Present Address: Ministry of Public Health, Doha, Qatar

**Keywords:** *Plasmodium vivax*, Genetic diversity, *Pvcsp*, *Pvs25*

## Abstract

**Background:**

Malaria is one of the important vector-borne diseases with high fatality rates in tropical countries. The pattern of emergence and spread of novel antigenic variants, leading to escape of vaccine-induced immunity might be factors responsible for severe malaria. A high level of polymorphism has been reported among malarial antigens which are under selection pressure imposed by host immunity. There are limited reports available on comparative stage-specific genetic diversity among *Plasmodium vivax* candidate genes in complicated vivax malaria. The present study was planned to study genetic diversity (*Pvcsp* and *Pvs25*) among complicated and uncomplicated *P. vivax* isolates.

**Methods:**

*Pvcsp* and *Pvs2*-specific PCRs and DNA sequencing were performed on *P. vivax* PCR positive samples. Genetic diversity was analysed using appropriate software.

**Results:**

The present study was carried out on 143 *P. vivax* clinical isolates, collected from Postgraduate Institute of Medical Education and Research, Chandigarh. Among the classic and variant types of *Pvcsp*, the VK210 (99%; 115/116) was found to be predominant in both complicated and uncomplicated group isolates. Out of the various peptide repeat motifs (PRMs) observed, GDRADGQPA (PRM1) and GDRAAGQPA (PRM2) was the most widely distributed among the *P. vivax* isolates. Whereas among the *Pvs25* isolates, 100% of double mutants (E97**Q**/I130**T**) in both the complicated (45/45) as well as in the uncomplicated (81/81) group was observed.

**Conclusion:**

An analysis of genetic variability enables an understanding of the role of genetic variants in severe vivax malaria.

## Background

Malaria is a vector-borne disease that poses serious threat to human life and is endemic throughout the tropics [1]. Classically severe malaria is known to be associated with *Plasmodium falciparum* [2]. Various studies across the globe have reported the presence of severe life-threatening symptoms in *Plasmodium vivax* patients [3–5]. Mechanisms underlying the biology, pathogenesis and epidemiology of severe vivax syndromes remain poorly understood and requires further investigation [6]. Several important parameters, such as the duration of the illness, resistance to particular drug, immunological crossreactivity, transmission by anopheline vectors, plays an important role in the constitution of *Plasmodium* species. The local and global epidemiology of a parasite species can only be estimated by distinguishing the different parasite strains circulating in the human host in endemic regions. Population and genetic diversity of *P. vivax* are important factors in understanding vivax malaria transmission dynamics [7]. Knowledge of the extent of genetic diversity enables the prediction of a pattern of emergence and spread of phenotypes of novel antigenic variants which leads to drug resistance or escape of vaccine-induced immunity, which might be responsible for the development of severe vivax malaria [8, 9]. Over the past few years extensive studies have been undertaken to understand the genetic diversity of *P. falciparum*, however there is a scarcity of literature on *P. vivax* genetic diversity [10].

Well-characterized polymorphic regions of both pre-erythrocytic and erythrocytic stage of *P. vivax* have been analysed to study genetic diversity patterns. For population genetics studies, circumsporozoite protein (CSP) (required in exo-erythrocytic cycle) is an important molecular marker to understand *P. vivax* diversity [11]. CSP is a prime target for anti-infection vaccines and has been studied extensively in terms of antigenicity and polymorphism. *Pvcsp* is a single copy gene encoding highly immunogenetic major sporozoite surface protein [8]. It encodes protein that consists of a central domain, having tandemly repeat sequences flanked by two non-repetitive conserved domains RI and RII: type I thrombospondin repeat (TSR) at C terminal and a 5-aa sequence at the N terminus as shown in Additional file 1: Figure S1A [11]. Three different genotypes (VK210, VK247, *P. vivax*-like) have been identified for the *Pvcsp* gene, based on the variation in the number of the peptide repeat motifs (PRMs) and sequences in the central repeat domain of *Pvcsp* [12]. The different genotypes are found to be globally distributed with geographic biases, where VK210 predominates in the endemic regions, while VK247 is reported from the regions, possessing cases of mixed infections [13, 14].

Several sexual stage antigens have been recognized on the basis of strong immunogenicity and potential transmission inhibiting activities [15]. The sexual stage antigens generally are of two types: 1st type consisting of post-fertilization antigens, such as *Pfs25* of *P. falciparum* and *Pvs25* and *Pvs28* of *P. vivax*, which are expressed on the zygote and ookinete surface; 2nd type are pre-fertilization antigens expressed on the both male and female gamete surface of malaria parasite, such as *Pfs48/45* and *Pfs230* [16]. *Pvs25* consists of a secretory N terminal signal sequence and 22 cysteine residues in a hydrophobic C terminus four epidermal growth factor (EGF) domains, as shown in Additional file 1: Figure S1B. These EGF domains consist of the consensus amino acid sequences of zygote/ookinete surface proteins of malaria parasites [17]. This protein plays an important role in various transformation processes occurring in the midgut of the mosquito. Polymorphism has also been reported in the *Pvs25* gene, like the other vaccine candidate genes, which can hamper the vaccine efficacy [18]. The present study was planned to study the molecular epidemiology, levels of genetic diversity (*Pvcsp and Pvs25*) among severe and non-severe *P. vivax* isolates collected from a tertiary hospital (PGIMER, Chandigarh) for a better understanding of the complexity of infection.

## Methods

### Study subjects

In the present study, a total of 143 *P. vivax*-positive patients attending PGIMER outpatient and inpatient departments (OPD and IPD) from adjoining states of Chandigarh (Haryana, Punjab, Uttar Pradesh) fulfilled the inclusion criteria of signs and symptoms of malaria and were enrolled for the period 2013 to 2016. The blood samples were collected aspectically by a trained practitioner and were transported to the laboratory for further processing. All the samples were further confirmed for *P. vivax* by molecular methods as described earlier by Kaur et al. [5]. WHO-based criteria for severe malaria was followed for the classification of *P. vivax*-positive patients into complicated and uncomplicated *P. vivax* groups.

### Amplification of *Plasmodium vivax* genetic diversity associated genes

The reference sequence of *P. vivax* (GU339059 and AF083502.1) was used for the primer designing of *P. vivax* genetic diversity genes *Pvcsp* and *Pvs25.* The sequences of primers used in the study are shown in Additional file 2: Table S1. The nested PCRs for *Pvcsp and Pvs25* genes were carried out on all *P. vivax*-positive samples. The negative control was included in each amplification reaction and precautions were taken to prevent cross-contamination. All PCR reaction mixtures were prepared using high fidelity Platinum Taq DNA polymerase (Thermo Fisher Scientific, Inc, Wilmington, DE, USA) shown in Additional file 2: Table S2. The thermal cycling profiles used for the amplification of *Pvcsp* and *Pvs25* are shown in Additional file 2: Table S3.

### Nucleotide sequencing and analysis

After visualization of the amplified products of *Pvcsp* and *Pvs25* on gel, the PCR products were purified using the PCR purification kit (Qiagen, Germany) as per manufacturer’s instruction. The nested PCR primers (NF and NR) for *Pvcsp* and *Pvs25* was then utilized to perform Sanger sequencing (Genewiz INC, NJ, USA) in forward and reverse directions for all the purified products. The obtained sequences were then edited and analysed to see the intraspecific variation (SNPs) if any, among the sequences [10]. To investigate the genetic diversity among the *Pvcsp* and *Pvs25* genes, MEGA vs 7.0.21 software was used [19]. The rate of synonymous and non-synonymous substitutions per site were estimated using the Nei and Gojobori method with Jukes and Cantor correction [20]. Mega software was used to test the null hypothesis (which states the strict neutrality of the gene), by estimating the dN−dS difference with the standard error of mean by 1000 bootstrap replications with two tail Z-test on the difference between dN and dS [21]. The rate of synonymous substitution is seen to accumulate at faster rate as compared to non-synonymous under the neutral model without effecting the parasite fitness (dS > dN). On the other hand, a high rate of non-synonymous substitutions is observed if positive selection is maintaining the polymorphism (dS < dN). A null hypothesis was assumed when the polymorphism was not under selection (dS = dN). To analyse the genetic relationship among the present study and worldwide haplotypes, Haplotype network was constructed [22].

## Results

In the present study, a total of 143 vivax malaria-positive patients were enrolled and demographic details and clinical history were collected at the time of sample collection (Table 1). The majority of *P. vivax* patients were from neighbouring states of Chandigarh: Haryana (29.4%), Punjab (23.7%) and Uttar Pradesh (21.7%). Out of 18.8% (27/143) cases of *P. vivax* observed from Chandigarh, the majority had travel history to the adjoining malarious regions of Chandigarh.

**Table 1:** Comparison of clinical features present in complicated and uncomplicated *P. vivax* infected patients

Variables	Complicated (n = 51) *n* (%) or mean ± SD	Uncomplicated (n = 76) *n* (%) or mean ± SD
Median age + SD	17 (8–27)	9.5 (4–20)
Male:Female	1:0.6	2:1
Fever (°F)	103 ± 1.2	103 ± 1.1
Chills and rigors	45 (88)	50 (66)
Nausea and vomiting	41 (80)	52 (69)
Duration (days)	7 ± 3	8 ± 5
Hb (g/dl)	8.9 ± 2.6	9.6 ± 2.0
Platelets/mm^3^	26,099 ± 20,073	70,564 ± 49,874
Urea (mg/dl)	54 ± 38	29 ± 15
Creatinine (mg/dl)	1.4 ± 1.4	0.6 ± 1.0
Total bilirubin (mg/dl)	2.13 ± 2.2	0.9 ± 0.4
AST/SGOT (IU/l)	73 ± 41	45 ± 21
ALT/SGPT (IU/l)	59 ± 34	37 ± 40

**Fig. 1:**
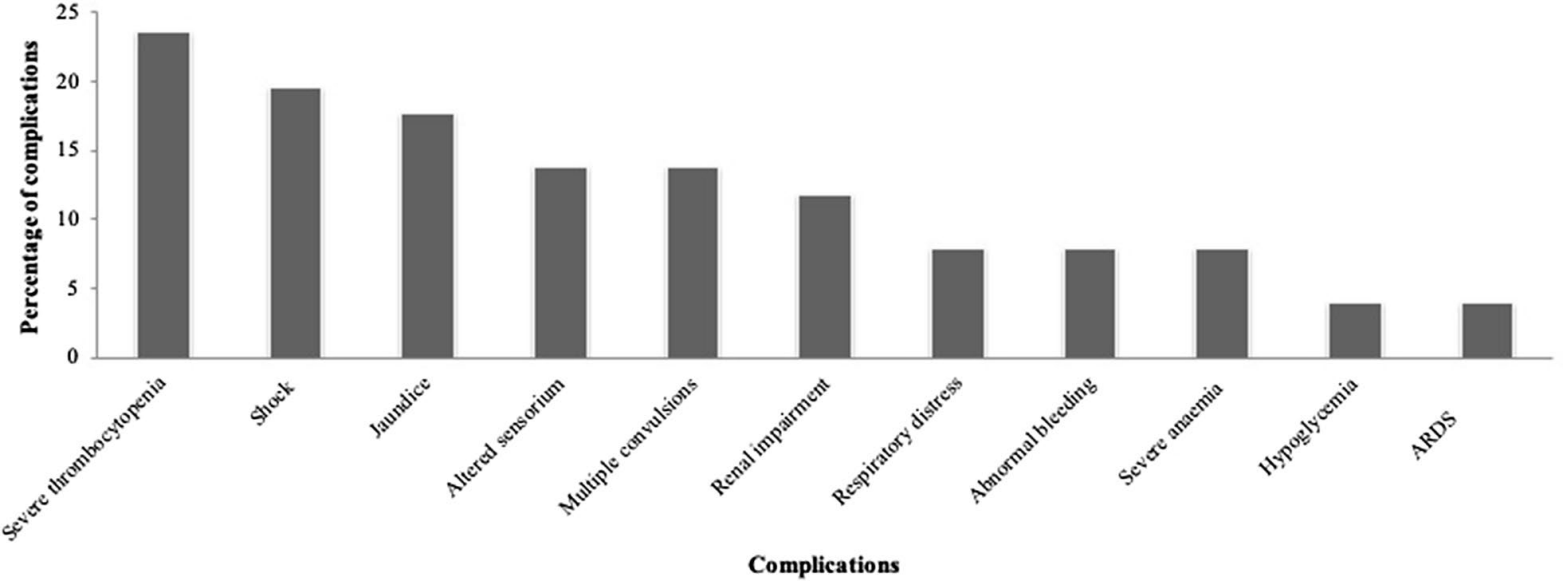
Frequency of complications among complicated vivax malaria (N = 51)

On the basis of WHO criteria for severe malaria, the patients were classified into two groups: complicated (35.7%; 51/143) and uncomplicated (64.3%; 92/143) [23]. The symptoms of fever accompanied with chills and rigors, headache, nausea, vomiting, and general body weakness was observed among the uncomplicated vivax malaria patients. Major complications seen to be present in the complicated group of patients were severe thrombocytopaenia (23.5%; 12/51), hypotensive shock or hypovolemic shock (19.6%; 10/51), jaundice (17.6%; 9/51), followed by the altered sensorium with the involvement of CNS (13.7%; 7/51), multiple convulsions (13.7%; 7/51) and renal impairment in 11.7% (6/51) of the patients, respectively (Fig. 1). Patients with single complication had severe thrombocytopaenia (23.5%; 12/51), jaundice (11.8%; 6/51), renal impairment (7.8%; 4/51), shock (5.9%; 3/51), and respiratory distress (5.9%; 3/51) as the major complications. The death of a 10 year old male child with history of 3 days’ illness was observed due to complications associated with malaria infection, such as seizure, hypotensive shock, nasal bleeding, febrile encephalopathy, and acute kidney injury with multiple organ dysfunction syndrome (MODS).

The nested PCR for *Pvcsp* and *Pvs25* was performed in a total of 143 *P. vivax* isolates. The sequencing of *Pvcsp* and *Pvs25* fragments was successful in a total of 81% (116/143) and 88% (126/143) of *P. vivax*-positive clinical isolates, respectively. The multiple sequence alignment (MSA) of the deduced protein sequence was performed as shown in Additional file 3: Figure S2.

### *Plasmodium vivax* circumsporozoite protein sequence analysis

**Fig. 2:**
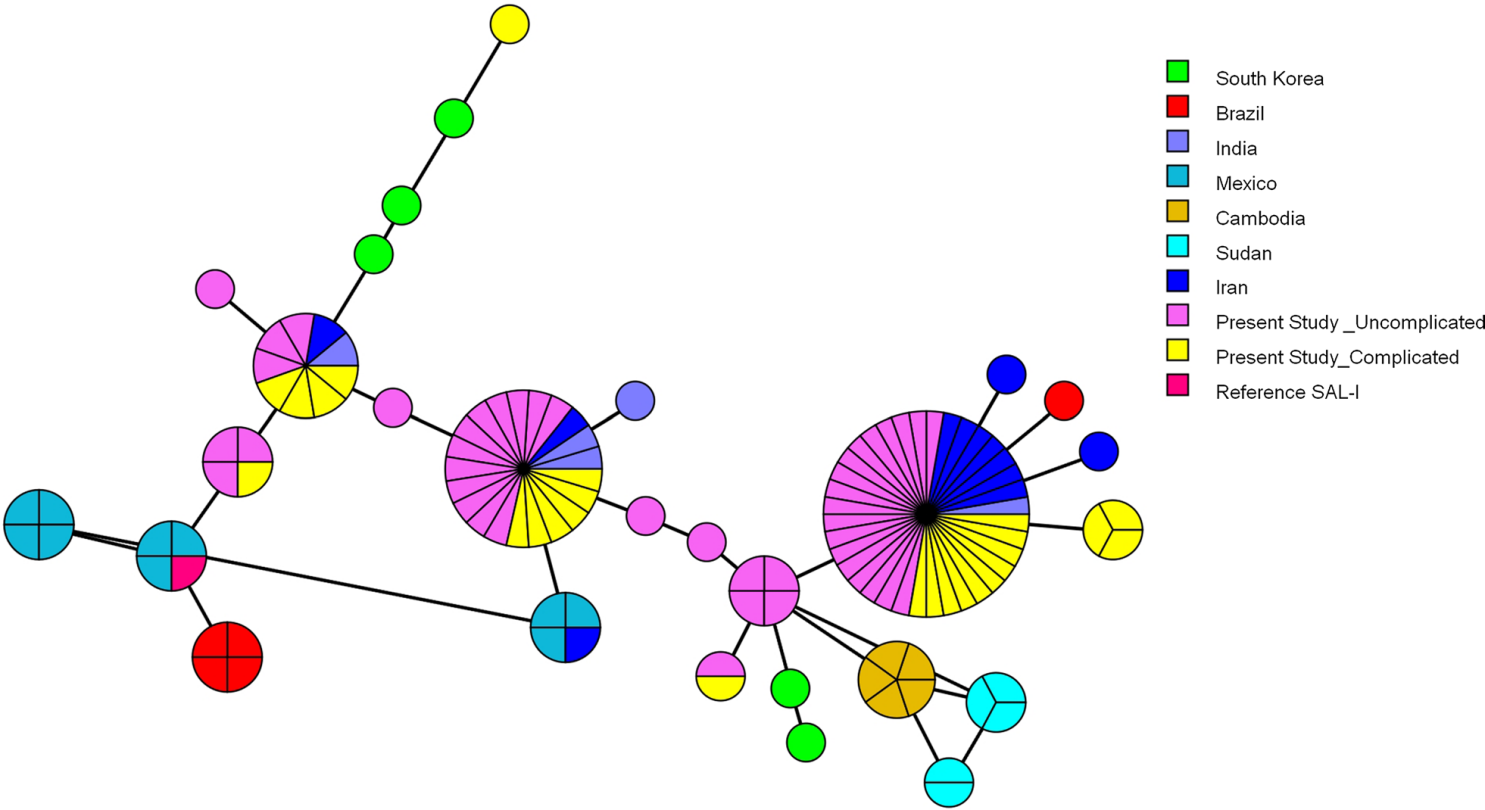
Median joining network of the *Plasmodium vivax Pvcsp* haplotypes. Branch lengths are proportional to divergence; node sizes are proportional to the total haplotype frequencies. The network shows 28 haplotypes found in 115 sequences. Every colour corresponds to a different geographic origin. Lines separating haplotypes represent mutational steps

The *Pvcsp* sequence analysis revealed the presence of only single infections of VK210 and VK247 variant types, without the presence of any mixed infection when compared to reference Sal-I sequence GU339059. The majority (99%; 115/116) of the *P. vivax* isolates were of VK210 variant type of *Pvcsp* and only one isolate was found of the VK247 variant type in the uncomplicated group. No *P. vivax* type of *Pvcsp* was identified in the clinical isolates. The 99% (115/116) of VK210 isolates consisted of variable repeats of majorly two PRMs, GDRADGQPA (PRM1), GDRAAGQPA (PRM2), followed by the conserved post-repeat sequence GNGAGGQAA (PRM4). Only one among 115 isolates was found to consist of third type of PRM (GDRAAGLPA) (PRM3). The KLKQP pre-repeat sequence was observed in all the clinical isolates of *Pvcsp*. Table 2 shows the observed non-synonymous substitution in the PRMs which gave rise to different PRMs types on the basis of different types of repeat allotypes (RATs). The rate of non-synonymous substitution per site was found to be higher in the complicated group of patients as compared to the synonymous substitution and uncomplicated group, leading to Dn–Ds of 0.003 ± 0.0010 SEM which was not found to be statistically significant (Table 3). The dN/dS ratio observed for *Pvcsp* in complicated group is > 1, which clearly suggests that the *Pvcsp* strains of this group are under positive selection compared to those of the uncomplicated group where the ratio was observed < 1. The haplotype network analysis shows the 28 haplotypes among the studied sequences (Fig. 2).

**Table 2:** Different nucleotide sequence of the repeat allotypes (RATs) and the peptide repeat motif is present in the central repeat region of *Pvcsp*

PRMs	Nucleotide sequence of the repeat allotypes (RATs)
GDRADGQPA (PRM1)	GGAGACAGAGCAGATGGACAGCCAGCA
	GGTGATAGAGCAGATGGACAGCCAGCA
	GGCGATAGAGCAGATGGACAGCCAGCA
	GGAGATAGAGCAGATGGACAACCAGCA
GDRAAGQPA (PRM2)	GGTGATAGAGCAGCTGGACAACCAGCA
	GGAGATAGAGCAGCTGGACAGCCAGCA
	GGAGATAGAGCAGCTGGACAACCAGCA
	GGAGATAGAGCAGCTGGACAGCCAGCA
	GGCGATAGAGCAGCTGGACAGCCAGCA
GDRAAGLPA (PRM3)	GGAGATAGAGCAGCTGGACTGCCAGCA
GNGAGGQAA (PRM4)	GGAAATGGTGCAGGTGGACAGGCAGCA

**Table 3:** Estimates of DNA sequence polymorphism in *Pvcsp* gene

	Transitions	Transversions	dS	dN	dN−dS ± SEM	Z test
*Pvcsp*						
Combined	0.008	0.016	0.028	0.024	− 0.004 ± 0.0011	dN/dS p = 0.35
Complicated	0.015	0.018	0.029	0.032	0.003 ± 0.0010	dN/dS p = 0.37
Uncomplicated	0.004	0.012	0.020	0.014	− 0.006 ± 0.0010	dN/dS p = 0.27

*dN* number of nonsynonymous substitutions, *dS* number of synonymous substitutions

### *Plasmodium vivax* sexual stage antigen 25 (*Pvs25*) sequence analysis

The *Pvs25* sequences of the clinical isolates were compared with the Sal-I reference strain (AF083502.1). The analysis revealed the presence of 100% of double mutant carrying the combination of E97**Q**/I130**T** in both the complicated (45/45) and uncomplicated (81/81) group of patients. The most frequent changes were observed in the EGF2 and EGF3 domain of *Pvs25*, where E97Q was found to be present in the EGF2 domain and I130T was present in the EGF3 domain of *Pvs25.* None of the SNPs was observed in the EGF1 and EGF4 domain of *Pvs25* (Table 4). The rate of non-synonymous and synonymous substitution in *Pvs25* was found to be zero in both groups of patients. This suggest that the *Pvs25* strains of this region are under constraint and are undergoing a purifying selection.

**Table 4:** *Pvs25* amino acid variation in present study and among worldwide isolates

Geographical regions	SS	EGF-1		EGF-2			EGF-3						EGF4		Refs
	0	0	0	0	0	0	1	1	1	1	1	1	1	1	
	0	3	3	7	8	9	3	3	3	3	3	4	7	7	
	2	5	8	9	7	7	0	1	2	7	8	9	0	4	
Sal-1 strain	N	L	M	C	Q	E	I	Q	S	C	A	K	C	E	
Present study	·	·	·	R	·	Q	T	·	·	·	·	·	·	·	
India	·	·	·	·	·	E/Q	T	Q/K	·	C/W	A/G	·	·	E/K	[33]
Iran	·	·	·	·	Q/K	E/Q	T	·	·	·	·	·	·	·	[18]
China	·	·	·	·	·	E/Q	T	·	·	·	·	·	·	·	[36]
China	·	L/M	·	·	·	E/Q	T	Q/K	·	·	·	·	·	·	[37]
Bangladesh	·	.	·	·	·	E/Q	T	Q/K	·	·	·	·	·	·	[24]
South Korea	N/D	·	·	·	·	E/Q	T	.	·	·	·	·	·	·	[25]
Thailand	·	·	·	·	·	E/Q	T	Q/K	·	·	·	·	·	·	[26]
Indonesia	·	·	·	·	·	Q	T	·	·	·	·	·	·	·	[27]
Mexico	·	·	·	·	Q/K	·	I/T	·	·	·	·	·	·	·	[28]

*SS* secretary signal sequence; *EGF* EGF-like domain

· Indicates identical amino acid residues compared to the Salvador I strain

## Discussion

Malaria is a disease of global importance and is one of the most important life-threatening parasitic infection affecting human beings. In the recent years, an upsurge of severe vivax malaria infection has been reported from various parts of the world, including India [24]. The genetically diverse population is thought to have increased potential to resist anti-malarials, vaccines and host immune response. Among the various genetic markers, *Pvcsp* is an important genetic marker used by many researchers from different geographical regions for elucidation of population genetics and evolutionary dynamics [8, 25, 26]. In the present study, the genetic diversity of *Pvcsp* was estimated among clinical isolates collected from PGIMER, Chandigarh. The results are in concordance with previously published results showing the prevalence of VK210 in 81–100% of isolates, emphasizing the dominance of VK210 over VK247 [6, 7, 9].

Variations present in the number of repeat units, along with differing amino acid and nucleotide sequence in repeat regions of *Plasmodium* antigens, are suggestive of natural selection pressure imposed by the host immune system [27]. In the present study, GDRADGQPA (PRM1) and GDRAAGQPA (PRM2) were found to be the two major PRMs. Earlier studies have reported the prevalence of these two major PRMs (GDRADGQPA, GDRAAGQPA) in clinical isolates [8, 28]. All the isolates were found to consist of similar pre-repeat sequence (KLKQP Region) and conserved post-repeat sequence GNGAGGQAA (PRM4). This conserved post-repeat sequence is found to present at the end of the sequence as a last unit in all the VK210 isolates, which was also reported in previous studies from Iran and Sri Lanka [8, 28]. One more peptide repeat motif GDRAAGLPA (PRM3) was observed at lower frequency (0.9%) among the isolates of the present study. The numbers of these PRMs are the main contributing factor for the development of genetic diversity in the *Pvcsp* gene among different geographical regions. The mode of evolution occurring in CSP of *P. vivax* is thought to be similar to that of CSP of *P. falciparum.* In *P. falciparum* it is known to include events of repeated non-reciprocal, intrahelical recombination events during mitotic DNA replication [29]. This phenomenon might lead to the generation of novel variants possessing the capability to evade the host immune response. Thus, the major factors responsible for the sequence evolution among the natural population involves the phenomenon of mitotic recombination accompanied with the positive selection of new variants in *P. vivax* [12]. Point mutations and intragenic recombination events might have also played a role in the generation of RATs, displaying remarkably similar arrangement in the RATs, suggestive of relatively recent origin from a common ancestor [12].

In the present study, the specific arrangement of the two dominant PRMs namely [GDRADGQPA (PRM1) and GDRAAGQPA (PRM2)] gave rise to a total of 28 different haplotypes in *Pvcsp.* The observed dN/dS ratio was found to be more than 1 in the complicated group of patients compared to the other group, which is suggestive of positive selection events occurring in the complicated group *P. vivax* isolates. The observed sequence variation in the VK210 between complicated and uncomplicated groups might be due to several factors, such as the distribution pattern of vector species (*Anopheles stephensi*, *Anopheles culicifacies*, *Anopheles subpictus*) and the difference in the infectivity of vector species by different genotypes of *Pvcsp* and/or host immunity against certain genotypes [30, 31].

*Pvs25* is one of the most promising vaccine candidate proteins among the cysteine-rich protein family. However, vaccine efficacy could be hindered due to reported antigenic diversity in *Pvs25* [32]. In the present study, the presence of 100% of double mutants carrying the combination of E97**Q**/I130**T** in both groups of patients was observed. The most frequent changes were observed in the EGF2 (E97**Q**) and EGF3 (I130**T**) domain of *Pvs25.* No novel amino acid substitution was observed in *Pvs25* in the present study. Chaurio et al. [22] reported overall low level of variation in *Pvs25*, with E97Q in 50% and I130T in 89% of the isolates, compared to present study results. The dominant prevalence of double amino acid substitutions at two positions (97**Q**/130**T**) observed in the present study was found to be similar to the previous study from Iran, having the prevalence of 97**Q**/130**T** (84%) among clinical isolates [18]. In contrast, a very high rate of non-synonymous amino acid substitution in *Pvs25* gene (n = 10 amino acid substitution) was observed by Prajapati et al. from India, however the presence of only two non-synonymous amino acid substitutions with double mutant haplotype was found in the present study [33]. Another study from China has reported the high prevalence of 97**Q** and 130**T** haplotype in 20% and 100% of the isolates, respectively [34]. The E97**Q** amino acid substitutions have been reported mainly from Asian isolates, i.e., from Bangladesh, Thailand, Indonesia, and South Korea [15, 33, 35–37], whereas the I130**T** amino acid have been mainly reported from both Asia and America [34]. The EGF2 and EGF3 of *Pvs25* were reported to consist of epitope recognition sites, identified for blocking antibodies [38]. As the sexual stage-specific proteins (*Pvs25*) are adapted to environment inside a vector’s body to complete its life cycle, the polymorphism observed among these surface antigens (*Pvs25*) has been associated with selective pressure exerted by the human immune system [39].

The results of the present study suggest the conserved nature of *Pvs25* compared to other vaccine candidate (*Pvcsp* and *PvdbpII*) in Chandigarh and its adjoining state regions, suggesting *Pvs25* is a better vaccine candidate gene. The specific expression of sexual stage proteins in the mosquito stages makes them able to avoid immune selection inside the human host, which might be the contributing factor for this conserved nature of *Pvs25* [34].

## Conclusion

Population genetic studies, such as in the present study, are required to understand the population genetic structure for the identification of signatures of balancing selection within *P. vivax* surface antigens. These studies will enable the identification of domains targeted by the host immune pressure and an understanding of the mechanism of host immune response for the identification of potential vaccine candidate [40]. A better understanding of genetic variability in different geographical regions will enlighten the role of these genetic variants in severe vivax malaria. These studies will be key for designing and implementing efficacious vaccines. The results of the present study will be used as baseline data for future studies.

## Supplementary information

**Additional file 1: Figure S1.** Schematic diagram of A) *Pvcsp* containing signal sequence (S), RI domain, central repeat region domain, post repeat region (PR), RII region containing thrombospondin repeat (TSR) and an anchor sequence; B) *Pvs25* containing signal sequence (SS), four EGF domains, and glycosylphosphatidylinositol (GPI) anchor.

**Additional file 2: Table S1.** Primers used for the amplification of *Pvcsp* and *Pvs25* genes. Table S2. Final concentration of PCR reagents used for nested and conventional PCRs of *Pvcsp* and *Pvs25.* **Table S3.** Thermal cycling profile used for the amplification of *Pvcsp* and *Pvs25* genes.

**Additional file 3: Figure S3.** Multiple sequence alignment (MSA) of A) *Plasmodium vivax* circumsporozoite protein (*Pvcsp*) and B) *Plasmodium vivax* sexual stage antigen *Pvs25* of the *Plasmodium vivax* clinical isolates using Clustal X 2.1.

## Data Availability

The datasets used and/or analysed during the current study are available from the corresponding author on reasonable request.

## References

[1] Wassmer SC, Taylor TE, Rathod PK, Mishra SK, Mohanty S, Arevalo-Herrera M (2015). Investigating the pathogenesis of severe malaria: a multidisciplinary and cross-geographical approach. Am J Trop Med Hyg.

[2] Sharma S, Aggarwal KC, Deswal S, Raut D, Roy N, Kapoor R (2013). The unusual presentation of a usual organism—the changing spectrum of the clinical manifestations of *Plasmodium vivax* malaria in children: a retrospective study. J Clin Diagn Res.

[3] Fabbri C, de Cássia Mascarenhas-Netto R, Lalwani P, Melo GC, Magalhães BML, Alexandre MAA (2013). Lipid peroxidation and antioxidant enzymes activity in *Plasmodium vivax* malaria patients evolving with cholestatic jaundice. Malar J.

[4] Kochar DK, Das A, Kochar SK, Saxena V, Sirohi P, Garg S (2009). Severe *Plasmodium vivax* malaria: a report on serial cases from Bikaner in northwestern India. Am J Trop Med Hyg.

[5] Kaur H, Sehgal R, Bansal D, Sultan AA, Bhalla A, Singhi SC (2018). Development of visually improved loop mediated isothermal amplification for the diagnosis of *Plasmodium vivax* malaria in a tertiary hospital in Chandigarh, North India. Am J Trop Med Hyg.

[6] Shabani SH, Zakeri S, Mehrizi AA, Mortazavi Y, Djadid ND (2016). Population genetics structure of *Plasmodium vivax* circumsporozoite protein during the elimination process in low and unstable malaria transmission areas, southeast of Iran. Acta Trop.

[7] Zhang L-L, Yao L-N, Chen H-L, Lu Q-Y, Ruan W (2018). Genetic diversity analysis of PvCSP and its application in tracking of *Plasmodium vivax*. Exp Parasitol.

[8] Dias S, Wickramarachchi T, Sahabandu I, Escalante AA, Udagama PV (2013). Population genetic structure of the *Plasmodium vivax* circumsporozoite protein (Pvcsp) in Sri Lanka. Gene.

[9] Talha AA, Pirahmadi S, Mehrizi AA, Djadid ND, Nour BYM, Zakeri S (2015). Molecular genetic analysis of *Plasmodium vivax* isolates from Eastern and Central Sudan using pvcsp and pvmsp-3alpha genes as molecular markers. Infect Genet Evol.

[10] Kaur H, Sehgal R, Goyal K, Makkar N, Yadav R, Bharti PK (2017). Genetic diversity of *Plasmodium falciparum* merozoite surface protein-1 (block 2), glutamate-rich protein and sexual stage antigen Pfs25 from Chandigarh, North India. Trop Med Int Health.

[11] Coppi A, Natarajan R, Pradel G, Bennett BL, James ER, Roggero MA (2011). The malaria circumsporozoite protein has two functional domains, each with distinct roles as sporozoites journey from mosquito to mammalian host. J Exp Med.

[12] Patil A, Orjuela-Sánchez P, da Silva-Nunes M, Ferreira MU (2010). Evolutionary dynamics of the immunodominant repeats of the *Plasmodium vivax* malaria-vaccine candidate circumsporozoite protein (CSP). Infect Genet Evol.

[13] Leclerc MC, Durand P, Gauthier C, Patot S, Billotte N, Menegon M (2004). Meager genetic variability of the human malaria agent *Plasmodium vivax*. Proc Natl Acad Sci USA.

[14] Zakeri S, Abouie Mehrizi A, Djadid ND, Snounou G (2006). Circumsporozoite protein gene diversity among temperate and tropical *Plasmodium vivax* isolates from Iran. Trop Med Int Health.

[15] Kang J-M, Ju H-L, Moon S-U, Cho P-Y, Bahk Y-Y, Sohn W-M (2013). Limited sequence polymorphisms of four transmission-blocking vaccine candidate antigens in *Plasmodium vivax* Korean isolates. Malar J.

[16] Tachibana M, Suwanabun N, Kaneko O, Iriko H, Otsuki H, Sattabongkot J (2015). *Plasmodium vivax* gametocyte proteins, Pvs48/45 and Pvs47, induce transmission-reducing antibodies by DNA immunization. Vaccine.

[17] Tsuboi T, Kaslow DC, Gozar MM, Tachibana M, Cao YM, Torii M (1998). Sequence polymorphism in two novel *Plasmodium vivax* ookinete surface proteins, Pvs25 and Pvs28, that are malaria transmission-blocking vaccine candidates. Mol Med.

[18] Zakeri S, Razavi S, Djadid ND (2009). Genetic diversity of transmission blocking vaccine candidate (Pvs25 and Pvs28) antigen in *Plasmodium vivax* clinical isolates from Iran. Acta Trop.

[19] Kumar S, Stecher G, Tamura K (2016). MEGA7: molecular evolutionary genetics analysis version 70 for bigger datasets. Mol Biol Evol.

[20] Nei M, Gojobori T (1986). Simple methods for estimating the numbers of synonymous and nonsynonymous nucleotide substitutions. Mol Biol Evol.

[21] Nei M, Kumar S (2000). Molecular evolution and phylogenetics.

[22] Chaurio RA, Pacheco MA, Cornejo OE, Durrego E, Stanley CE, Castillo AI (2016). Evolution of the transmission-blocking vaccine candidates Pvs28 and Pvs25 in *Plasmodium vivax*: geographic differentiation and evidence of positive selection. PLoS Negl Trop Dis.

[23] WHO (2012). Management of severe malaria—a practical handbook.

[24] Nandwani S, Pande A, Saluja M (2014). Clinical profile of severe malaria: study from a tertiary care center in north India. J Parasit Dis.

[25] Kibria MG, Elahi R, Mohon AN, Khan WA, Haque R, Alam MS (2015). Genetic diversity of *Plasmodium vivax* in clinical isolates from Bangladesh. Malar J.

[26] Raza A, Ghanchi NK, Thaver AM, Jafri S, Beg MA (2013). Genetic diversity of *Plasmodium vivax* clinical isolates from southern Pakistan using pvcsp and pvmsp1 genetic markers. Malar J.

[27] Hughes AL (2004). The evolution of amino acid repeat arrays in *Plasmodium* and other organisms. J Mol Evol.

[28] Zakeri S, Mehrizi AA, Mamaghani S, Noorizadeh S, Snounou G, Djadid ND (2006). Population structure analysis of *Plasmodium vivax* in areas of Iran with different malaria endemicity. Am J Trop Med Hyg.

[29] McConkey GA, Waters AP, McCutchan TF (1990). The generation of genetic diversity in malaria parasites. Annu Rev Microbiol.

[30] Zakeri S, Raeisi A, Afsharpad M, Kakar Q, Ghasemi F, Atta H (2010). Molecular characterization of *Plasmodium vivax* clinical isolates in Pakistan and Iran using pvmsp-1, pvmsp-3alpha and pvcsp genes as molecular markers. Parasitol Int.

[31] Zakeri S, Safi N, Afsharpad M, Butt W, Ghasemi F, Mehrizi AA (2010). Genetic structure of *Plasmodium vivax* isolates from two malaria endemic areas in Afghanistan. Acta Trop.

[32] Prajapati SK, Joshi H, Dua VK (2011). Antigenic repertoire of *Plasmodium vivax* transmission-blocking vaccine candidates from the Indian subcontinent. Malar J.

[33] Han ET, Lee WJ, Sattabongkot J, Jang JW, Nam MH, An SSA (2010). Sequence polymorphisms of *Plasmodium vivax* ookinete surface proteins (Pvs25 and Pvs28) from clinical isolates in Korea. Trop Med Int Health.

[34] Feng H, Zheng L, Zhu X, Wang G, Pan Y, Li Y (2011). Genetic diversity of transmission-blocking vaccine candidates Pvs25 and Pvs28 in *Plasmodium vivax* isolates from Yunnan Province, China. Parasit Vectors.

[35] Tsuboi T, Kaneko O, Cao Y-M, Tachibana M, Yoshihiro Y, Nagao T (2004). A rapid genotyping method for the vivax malaria transmission-blocking vaccine candidates, Pvs25 and Pvs28. Parasitol Int.

[36] Sattabongkot J, Tsuboi T, Hisaeda H, Tachibana M, Suwanabun N, Rungruang T (2003). Blocking of transmission to mosquitoes by antibody to *Plasmodium vivax* malaria vaccine candidates Pvs25 and Pvs28 despite antigenic polymorphism in field isolates. Am J Trop Med Hyg.

[37] Escalante AA, Cornejo OE, Freeland DE, Poe AC, Durrego E, Collins WE (2005). A monkey’s tale: the origin of *Plasmodium vivax* as a human malaria parasite. Proc Natl Acad Sci USA.

[38] Saxena AK, Singh K, Su H-P, Klein MM, Stowers AW, Saul AJ (2006). The essential mosquito-stage P25 and P28 proteins from *Plasmodium* form tile-like triangular prisms. Nat Struct Mol Biol.

[39] Escalante AA, Cornejo OE, Rojas A, Udhayakumar V, Lal AA (2004). Assessing the effect of natural selection in malaria parasites. Trends Parasitol.

[40] Arnott A, Barry AE, Reeder JC (2012). Understanding the population genetics of *Plasmodium vivax* is essential for malaria control and elimination. Malar J.

