# Supplementary figures and images for: Exploration of genetic diversity of Plasmodium vivax circumsporozoite protein (Pvcsp) and Plasmodium vivax sexual stage antigen (Pvs25) among North Indian isolates
Source: Malar J. 2019 Sep 6;18:308. doi: 10.1186/s12936-019-2939-z (PMC6731556; doi:10.1186/s12936-019-2939-z)

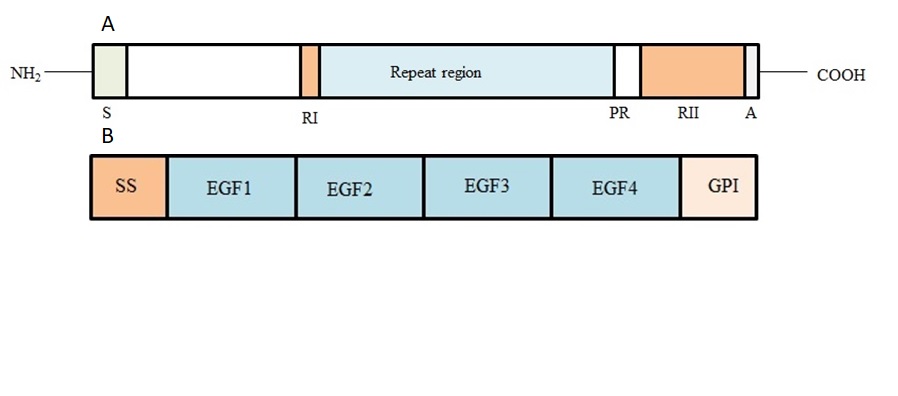

Supplement: Supplementary file 1 — Additional file 1: Figure S1. Schematic diagram of A) Pvcsp containing signal sequence (S), RI domain, central repeat region domain, post repeat region (PR), RII region containing thrombospondin repeat (TSR) and an anchor sequence; B) Pvs25 containing signal sequence (SS), four EGF domains, and glycosylphosphatidylinositol (GPI) anchor. [file 12936_2019_2939_MOESM1_ESM.jpg]

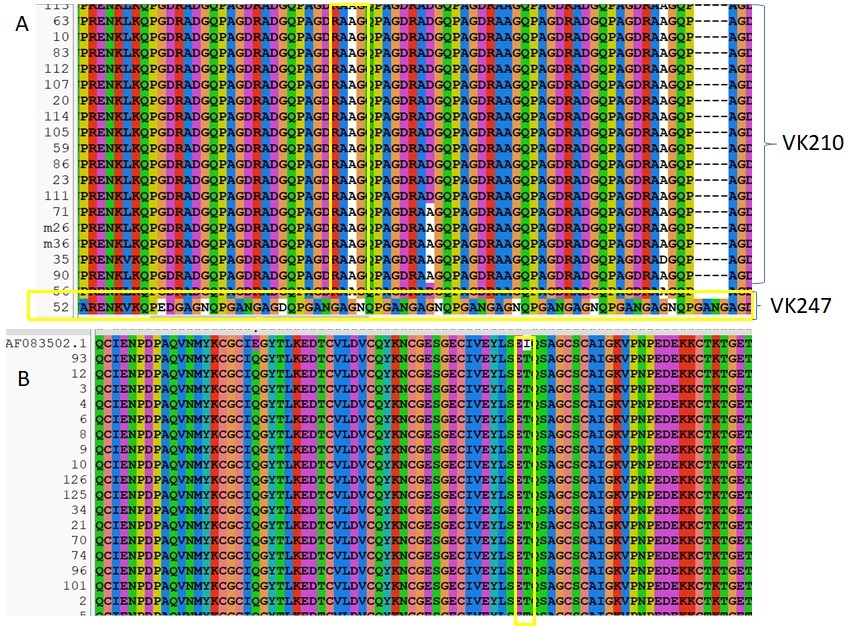

Supplement: Supplementary file 3 — Additional file 3: Figure S3. Multiple sequence alignment (MSA) of A) Plasmodium vivax circumsporozoite protein (Pvcsp) and B) Plasmodium vivax sexual stage antigen Pvs25 of the Plasmodium vivax clinical isolates using Clustal X 2.1. [file 12936_2019_2939_MOESM3_ESM.jpg]
