# Supplementary material for: Exploration of genetic diversity of Plasmodium vivax circumsporozoite protein (Pvcsp) and Plasmodium vivax sexual stage antigen (Pvs25) among North Indian isolates
Source: Malar J. 2019 Sep 6;18:308. doi: 10.1186/s12936-019-2939-z (PMC6731556; doi:10.1186/s12936-019-2939-z)
Supplement: Supplementary file 2 — Additional file 2: Table S1. Primers used for the amplification of Pvcsp and Pvs25 genes. Table S2. Final concentration of PCR reagents used for nested and conventional PCRs of Pvcsp and Pvs25. Table S3. Thermal cycling profile used for the amplification of Pvcsp and Pvs25 genes. [file 12936_2019_2939_MOESM2_ESM.doc]

**Table S1: Primers used for the amplification of *Pvcsp* and *Pvs25*** genes

| **Genes** | **Primers** | **Sequence (5’-3’)** | **Product size (bp)** |
| --- | --- | --- | --- |
| ***Pvcsp*** | Outer forward PF | ATGTAGATCTGTCCAAGGCCATAAA | 1064 |
|  | Outer reverse PR | TAATTGAATAATGCTAGGACTAACAATATG |  |
|  | Inner forward NF | GCAGAACCAAAAAATCCACGTGAAAATAAG | 686 |
|  | Inner reverse NR | CCAACGGTAGCTCTAACTTTATCTAGGTAT |  |
| ***Pvs25*** | Outer forward PF | CTTTAGTCCTTTCTCTTCTC | 895 |
|  | Outer reverse PR | AGGATGTTAAATACAGAATAGG |  |
|  | Inner forward NF | GACTTTCGTTTCACAGCACT | 810 |
|  | Inner reverse NR | CGTAAAGCCTTCCATACACTG |  |

***Table S2: Final concentration of PCR reagents used for nested and conventional PCRs of Pvcsp and*** Pvs25

| **Genes** | **Primers** |  | **PCR buffer**  **10X** | **MgSO_4_50mM** | **Primers 10µM** | **dNTPs 10mM** | **Taq polymerase 5U/µL** | **DNA** | **Final volume**  **(nuclease free water) µL** | |
| --- | --- | --- | --- | --- | --- | --- | --- | --- | --- | --- |
| ***Pvcsp*** | PF | Nest1 | 1X | 2.5 | 0.5 | 0.8 | 0.5 | 2 | 12.5 |  |
|  | PR |  |  |  |  |  |  |  |  |  |
|  | NF | Nest2 | 1X | 2 | 0.4 | 0.8 | 0.5 | 2(1:10) | 25 |  |
|  | NR |  |  |  |  |  |  |  |  |  |
| ***Pvs25*** | PF | Nest1 | 1X | 2 | 0.8 | 0.8 | 1 | 2 | 12.5 |  |
|  | PR |  |  |  |  |  |  |  |  |  |
|  | NF | Nest2 | 1X | 3 | 0.8 | 0.8 | 1 | 2(1:10) | 25 |  |
|  | NR |  |  |  |  |  |  |  |  |  |

**Table S3: Thermal cycling profile used for the amplification of *Pvcsp* and *Pvs25*** genes

| **Genes** |  |  | **1^st^round (⁰C/minute)** | | **2^nd^ round (⁰C/minute)** | |
| --- | --- | --- | --- | --- | --- | --- |
| ***Pvcsp*** | Stage 1 | Initial denaturation | 94/10 | | 94/10 | |
|  | Stage 2 | Denaturation | 94/01:00 | 30 rounds | 94/01:00 | 35 rounds |
|  |  | Annealing | 58/01:00 |  | 60/01:30 |  |
|  |  | Polymerization | 72/01:30 |  | 72/01:00 |  |
|  | Stage 3 | Final extension | 72/10 | | 72/10 | |
| ***Pvs25*** | Stage 1 | Initial denaturation | 94/10 | | 94/10 | |
|  | Stage 2 | Denaturation | 94/01:00 | | 94/00:30 | |
|  |  | Annealing | 49/01:00 35 rounds | | 53/01:00 30 rounds | |
|  |  | Polymerization | 72/01:00 | | 72/01:00 | |
|  | Stage 3 | Final extension | 72/10 | | 72/10 | |
